# Strategies to Increase Testosterone in Men Seeking Fertility

**DOI:** 10.5152/tud.2020.20436

**Published:** 2023-01-01

**Authors:** Kevin Y. Chu, Justin K. Achua, Ranjith Ramasamy

**Affiliations:** 1 Department of Urology, University of Miami Miller School of Medicine, Miami, FL, USA

**Keywords:** Hypogonadism, infertility, testosterone, testosterone therapy

## Abstract

Prevalence of testosterone deficiency is increasing in the adolescent and young adult male population. As the average paternal age rises, there is a significant population of men with hypogonadism seeking testosterone therapy wishing to achieve or maintain fertility potential. Identification of potential lifestyle modifications that may improve the testosterone deficiency is one of the initial interventions of the holistic strategy in treatment. This is followed by drug therapy; however, traditional testosterone therapy acts as a contraceptive by suppressing the hypothalamus-pituitary-gonadal axis and therefore cannot be used as a treatment strategy. A solution has been the off-label use of selective estrogen receptor modulators, human chorionic gonadotropin, and anastrozole inhibitors to treat hypogonadal symptoms while increasing intratesticular testosterone, a necessity for spermatogenesis. Recently, a novel therapy, Natesto intranasal testosterone gel, has been shown to increase serum testosterone levels while maintaining semen parameters. This is hypothesized to be because of its short-acting properties having lesser effect on the hypothalamus-pituitary-gonadal axis, in contrast to the long-acting properties of traditional testosterone therapy. It is important to differentiate hypogonadal men between those seeking to achieve or maintain fertility status because the drug therapy of choice differs. This can be accomplished by determining the levels of 17-hydroxyprogesterone, because it is a biomarker for intratesticular testosterone. Those with low 17-hydroxyprogesterone may wish to initiate treatment with alternative therapies, whereas those with high 17-hydroxyprogesterone may trial short-acting testosterone therapies. As the urologist’s armamentarium continues to increase, better strategies to increase testosterone levels in men seeking fertility can be achieved.

**Main Points**

- Selective estrogen receptor modulators, hypothalamic- pituitary-gonadal (hCG), and anastrozole inhibitors are used off-label to increase intratesticular testosterone, which is essential for spermatogenesis.
- Short-acting testosterone preparations, such as Natesto intranasal gel, has been shown to increase serum testosterone levels while maintaining semen parameters in a clinical trial. The short-acting properties are hypothesized to have lesser effect on the HPG axis than traditional long-acting testosterone therapy.
- 17-Hydroxyprogesterone is a biomarker for intratesticular testosterone, and its levels can be used to differentiate between men with hypogonadism needing improvement versus those who need to maintain their semen parameters.

## Introduction

Male hypogonadism is estimated to have a prevalence of 2.1% and 5.6% by the Massachusetts Male Aging Study and the European Male Aging Study, respectively.^1,2^ A diagnosis of male hypogonadism is defined as a state of low serum testosterone (>300 ng/dL) and concurrent clinical symptoms that impact physical and mental health.^3^ The symptoms are broad and vary depending on the age of onset and severity of testosterone deficiency. These symptoms include decreased libido, erectile dysfunction, infertility, low energy, low mood, depression, gynecomastia, decreased lean muscle mass, increased body fat gain, and osteoporosis.^3,4^ Low serum testosterone has also been shown to have potentially long-term negative effects on cardiovascular health, metabolism, and longevity.^5^ Because the average paternal age has increased over time, and hypogonadism rates increase with age, the challenge of treating low testosterone while maintaining fertility status has become paramount.^6^ Although testosterone deficiency was commonly thought to be a diagnosis in only older men, recent studies have started to show an increase in prevalence in younger men too.^7^ This impacts the potential testosterone therapy (TT) plan because the patient’s desire for conception needs to be considered.

Testosterone deficiency in adolescents and young adult (AYA) men, defined as those between the ages of 15-39, has a prevalence of approximately 20%. In addition, studies have shown that total testosterone levels among AYA men have been declining over the past few decades. Although increasing obesity rates have shown to be an independentpredictor of declining testosterone levels, Lokeshwar et al^7^ demonstrated a decline in total testosterone among AYA males despite controlling for confounders. Adolescents and young adult men diagnosed with hypogonadism present with initial symptoms of low energy and fatigue, in contrast to the complaints of decreased libido or erectile dysfunction in older men.^8^ Among this patient population, strategies to increase testosterone levels to address hypogonadal symptoms must be well thought out to maintain fertility status.

Clinical evaluation of hypogonadism can be assisted with the use of various questionnaires such as the Androgen Deficiency in Aging Male, the Aging Male Survey, and the Massachusetts Male Aging Study.^9^ For laboratory evaluation, it is suggested that measurement of serum testosterone levels be performed before 10:00 AM, because the testosterone levels peak at that time.^10^ If the measurement of total testosterone is near the lower limit of normal, then the free or bioavailable testosterone may be measured for a suspected alteration in sex hormone binding globulin. Further assessment of serum luteinizing hormone (LH) and follicle-stimulating hormone (FSH) can differentiate between primary and secondary hypogonadism.^11^ This difference in the classification of primary and secondary hypogonadism is important in determining which therapies will work to help increase testosterone levels.

Primary and secondary hypogonadism are distinguished by the deficiency in the level of the hypothalamic-pituitary-gonadal (HPG) axis. Secondary hypogonadism is the most common form of male hypogonadism and is defined as a failure to produce hormones at the level of the hypothalamus or the pituitary gland. As a result, there are insufficient hormones to stimulate testosterone production in the testes.^12^ Serum laboratory test results typically show decreased testosterone and LH levels. Secondary hypogonadism is associated with conditions such as prolactinoma, pituitary tumors, hemochromatosis, and genetic conditions. In contrast, primary hypogonadism is defined as a failure of testosterone production within the testes itself. Serum LH is elevated because of the lack of testosterone, causing negative feedback on the hypothalamus and pituitary gland. Genetic disorders, testicular trauma, or infections can lead to primary hypogonadism.^3^ Compensated hypogonadism is a more recent clinical classification that is defined as a prodromal state of primary hypogonadism in which serum LH levels are elevated to compensate and maintain testosterone levels within the normal range.^12^

Because male hypogonadism revolves around the abnormalities of the HPG axis, it is important to have primer knowledge on this topic when planning for therapy strategies to increase testosterone levels. In this classic regulated feedback loop, pituitary gonadotropins (LH and FSH) are released in response to the pulsatile release of the gonadotropin releasing hormone (GnRH) by the hypothalamus. LH binds to Leydig cells and stimulates the release of testosterone in the testes. FSH binds to Sertoli cells and supports spermatogenesis. The actions of LH and FSH work in conjunction as testosterone stimulates sperm production and the development of secondary sex characteristics in men. Hormonal levels are maintained in homeostasis by neg-ative feedback caused by testosterone and inhibin B.^4^ The HPG axis is responsible for the secretion of the male hormone testosterone and maintaining normal spermatogenesis, and this is important to keep in mind when planning treatment for men with hypogonadism.

In this review, we have summarized the various therapies available for increasing testosterone levels in men seeking fertility. These therapies include selective estrogen receptor modulators, gonadotropins, aromatase inhibitors, and TT. In addition, it is important to consider non-drug approaches to increase testosterone levels. This allows for a holistic approach to treating hypogonadism. Treating hypogonadism can be difficult for physicians, because inappropriate therapies, such as exogenous testosterone, will suppress spermatogenesis and impact future fertility. The physician and the patient must have a thoughtful discussion before initiating the treatment.

## Drug Therapies for Testosterone Deficiency

### Testosterone Therapy

Testosterone therapy is available in various formulations with differing methods of administration. These delivery methods include transdermal, buccal, injectable, and subcutaneous preparations. These therapies, considered exogenous testosterone, may act as a male contraceptive and impact men with hypogonadism seeking to maintain fertility status.^13^ One of the properties of TT, which is being closely researched, is the effect of short-acting compared with long- acting preparations on the HPG axis. Long-acting formulations are hypothesized to cause inhibition of the HPG axis through a steady negative feedback, whereas short-acting formulations have a lesser effect on the pulsatile release of GnRH^14^ resulting in lesser effect on the HPG axis and thus preserving the downstream effects needed for fertility. Masterson et al^15^ performed a systematic review of 8 studies, which included 793 men with hypogonadism and their respective FSH and LH levels following TT. They found that short-acting testosterone preparations had lesser effect on FSH and LH suppression than long-acting formulations. Furthermore, Chu et al^16^ studied the effects of short-acting and long-acting exogenous testosterone in a mouse-breeding study and observed similar fertility potential between mice receiving short-acting testosterone and mice receiving placebo injections. However, the mice receiving long-acting testosterone had impaired reproductive capacity.

Natesto, a nasal testosterone gel preparation (Acerus Pharmaceu-ticals, Mississauga, Canada), received Food and Drug Administra-tion (FDA) approval in 2014.^17^ Natesto is considered a short-acting testosterone preparation owing to its half-life and requires b.i.d. or t.i.d. dosing to reach normal levels of testosterone in men with hypogonadism as evidenced in a randomized study by Rogol et al.^18^ The intranasal delivery system avoids the usual TT side effects of injection site reactions, dermatitis, or transference. Great bioavailability is obtained through the high permeability ofthe nasal mucosa. Clinical studies showed rapid absorption and serum concentrations reaching maximum levels at 60 minutes post administration.^19^ Rhinorrhea, sore throat, and nasal crustiness were most common reported drug- related side effects.^18^

Ramasamy Ranjith et al^20^ performed an open label, single arm clinical trial on Natesto and studied the effect of Natesto on reproductive hormones, semen parameters, and hypogonadal symptoms. They enrolled 60 men with hypogonadism and administered the treatment for 6 months. At the end of the study, 90.9% of men had achieved normal testosterone levels, and 81.8% and 72.7% had preserved FSH and LH levels, respectively. In addition, 93.9% of the men had total motile sperm count greater than 5 million at the endpoint of the study. Although further study is required, Natesto nasal testosterone gel remains an attractive option for men with hypogonadism interested in fertility.

Most recently, Jatenzo (Clarus Therapeutics, Northbrook, IL, USA) became the first oral formulation of testosterone to be approved by the FDA.^21^ Oral formulations of testosterone have been available for the past 50 years, but poor bioavailability secondary to first-pass metabolism and hepatotoxicity have limited its approval for use in patients.^11^ There were also concerns for possible increased cardiovascular risk with increased low-density lipoprotein and decreased high-density lipoprotein levels.^22^ Testosterone undecanoate (TU) is an ester form that was favored for research owing to its lipophilic properties. Testosterone undecanoate is absorbed into the intestinal lymphatics and bypasses hepatic processing.^23^ Jatenzo, an oral TU formulation, is taken on a b.i.d. dosing schedule and carries shortacting testosterone properties.^21^ Although its current effect on fertility is unknown, further research in the future may prove this to be another useful TT to maintain male reproductive potential.

### Alternative Therapy: Selective Estrogen Receptor Modulators (SERMs)

Selective estrogen receptor modulators (SERMs) are a class of drugs that have an agonistic or antagonistic effect on estrogen receptors depending on the receptor location.^24^ Clomiphene citrate was the first SERM approved in the USA for ovulation induction therapy in infertile women in 1967.^25^ Since then, the therapy has been used to treat testosterone deficiency as a non-FDA approved off-label drug.^26^ Clomiphene citrate improves serum testosterone through competitive inhibition of estrogen receptors in the hypothalamus and pituitary. Antagonism of these receptors prevents the negative feedback loop on the HPG axis by circulating estrogens resulting in the rise of levels of LH and FSH from the pituitary and subsequently increasing endogenous serum testosterone production.^27^

The route of administration for clomiphene citrate is oral. Typical therapy for male hypogonadism with clomiphene citrate starts at 25 mg every other day and can be titrated up to 50 mg daily to meet the goal testosterone levels. The half-life is approximately 10 hours, and side effects include gynecomastia and water retention.^28,29^ Clo- miphene citrate is a popular drug to treat male hypogonadism in men seeking fertility because of its minimal side effect profile, low consumer cost, simplicity as an oral medication, and the ability for men to maintain fertility.

### Alternative Therapy: Gonadotropins

Patients with secondary hypogonadism can be treated with a gonadotropin replacement therapy in addition to treating the underlying disease process. This class of drugs directly replaces the physiologic effects of endogenous gonadotropins within the HPG axis to promote testosterone production. The hypothalamus releases GnRH in a pulsatile fashion to stimulate the pulsatile release of LH and FSH from the anterior pituitary. Gonadotropin releasing hormone or LH can be supplemented to increase endogenous testosterone production in the absence or disruption of upstream components of the HPG axis.^30^ Jacobson et al^31^ in 1979 first published GnRH as a therapeutic modality. Doses of 5-20 ng are given every 1-2 hours, via a subcutaneous portable infusion pump, with normal testosterone levels reached within 18-24 months in most men. The subcutaneous portable infusion pumps mimic the normal pulsatile release of GnRH; however, this treatment modality is used strictly for research purposes because of the inconvenience of having to wear a pump and requiring constant dosing.^32^

Normally, LH stimulates testosterone production in the testes; how-everhuman chorionic gonadotropin (hCG), an analogue of LH, is used for its similar activity at the LH receptor; hCG is typically admin-istered as a subcutaneous injection in 1500-3000 IU equivalents 1-3 times weekly.^32^ The side effect profiles of GnRH and hCG are minimal with gynecomastia most commonly reported. Similar to SERMs, gonadotropins may protect fertility and spermatogenesis with their maintenance of intratesticular testosterone production.^33^

### Alternative Therapy: Aromatase Inhibitors

Aromatase inhibitors (AIs) anastrozole and letrozole increase endogenous testosterone production via the inhibition of the peripheral conversion of testosterone (T) to estradiol (E) by the aromatase enzyme. This has the additional effect of reducing the negative feedback on the HPG axis by reducing serum estrogens and increasing endogenous gonadotropin levels.^34^ In men, a T/E ratio of >10 is considered normal. The use of AIs, such as anastrozole 1 mg daily or letrozole 2.5 mg daily, can restore the T/E ratio to normal, thereby improving hypogonadism and subfertility.^35,36^ Aromatase inhibitors have also been shown to be useful in the correction of elevated estradiol levels associated with TT. The use of AIs is considered off-label for the treatment of hypogonadism or elevated estradiol levels. Aromatase inhibitors rarely cause nausea, decreased libido, and decreased bone mineral density and are generally well tolerated.^37^ Estradiol has been shown to play an essential role in bone health and libido in men, and suppression of estradiol to undetectable levels can impact these functions. With this in mind, the dosing of anastrozole at 1 mg twice weekly or 3 times weekly may be sufficient to correct abnormal T/E ratios.^38^

### Differentiating Treatment with Alternative Therapies Versus Novel Testosterone Therapy

One of the critical components of hypogonadal treatment in males seeking fertility is identifying if the semen parameters need to be improved, or if they just need to be maintained. Intratesticular tes-tosterone is increased by the alternative therapies of SERMs, hCG, and anastrozole inhibitors. In contrast, exogenous TT causes a suppression and resulting decrease in intratesticular testosterone levels. Intratesticular testosterone is a necessity for spermatogen-esis to occur, yet until recently there were no serum biomarkers to quantify levels. Quantification could only occur with testicular biopsy and/or aspiration.^39,40^ Recent studies have shown the possibility of using 17-hydroxyprogesterone (17-OHP) as a biomarker for intra- testicular testosterone. This biomarker not only provides for ease of access in studying the effects of various intratesticular testosterone levels on spermatogenesis but also allows for fine-tuning of alternative testosterone therapies. Lima et al^41^ performed a cross-sectional analysis which showed undetectable 17-OHP in men on exogenous therapy compared with increased 17-OHP in men on hCG or clomi- phene citrate. This biomarker may also lend itself to identifying men with adequate intratesticular testosterone prior to TT, and thus it is advisable to initiate treatment with short-acting TT to maintain their semen parameters instead of alternative testosterone therapies. Further research on 17-OHP is required, but it does show great promise in an area requiring a dependable biomarker.

### Non-drug Strategies for Testosterone Deficiency

It is important to consider non-drug therapies for a holistic treat-ment of patients with testosterone deficiency seeking to procreate. Lifestyle modifications such as diet, exercise, weight loss, sleep improvement, and stress reduction have been shown to possibly increase testosterone levels.^42^ It was long considered that a plantbased diet was linked to lower testosterone levels, but recent findings by Kuchakulla et al^43^ showed that, unlike body mass index (BMI) and age, there was no association between a healthy plant-based diet and serum testosterone levels. Fantus et al^44^ found that men who practice a low-fat diet had lower serum testosterone levels than men with non-restrictive diets. This relationship between low-fat diet and testosterone remained true even when comorbidities, age, BMI, and activity levels were controlled. Multiple studies have compared testosterone levels of obese men prior to and after bariatric surgery. Obese men were found to have lower testosterone levels before they underwent bariatric surgery. Studies on the results of semen parameters were inconsistent to enable any conclusions.^45,46^ Along with drug therapy for testosterone deficiency treatment, non-drug therapies should be also be a part of the broad treatment strategy, because they may act in a synergistic manner and advocate for an overall healthier lifestyle as per the American Urological Association guidelines.

## Conclusion

Testosterone deficiency is a clinical problem that is increasing in prevalence among AYA. As the average paternal age increases, the hypogonadal population seeking to improve or maintain fertility gets larger. A holistic approach with counseling on lifestyle modifications is a must. Alternative therapies such as SERMs, hCG, or anastrozole inhibitors have been used off-label for treatment. Recent drug development of a short-acting testosterone formulation, Natesto, has been approved for TT, and studies have shown maintenance of semen parameters with it. In addition, 17-OHP has been identified as a potential biomarker for intratesticular testosterone levels, which will allow for better counseling of patients on the appropriate treatment strategy. As the urologist’s armamentarium continues to increase, better strategies to increase testosterone levels in men seeking fertility can be achieved.
